# Clinical application of Vitek-derived minimum inhibitory concentration values: Proof of concept study

**DOI:** 10.4102/sajid.v38i1.498

**Published:** 2023-03-31

**Authors:** Warren Lowman

**Affiliations:** 1 Department of Clinical Microbiology, PathCare/Vermaak, Johannesburg, South Africa; 2 Department of Clinical Microbiology and Infection Prevention and Control, Wits Donald Gordon Medical Centre, Johannesburg, South Africa; 3 Department of Clinical Microbiology and Infectious Diseases, Faculty of Health Sciences, University of the Witwatersrand, Johannesburg, South Africa

**Keywords:** AST, MIC, ECOFF, Vitek, clinical application

## Abstract

**Background:**

Minimum inhibitory concentration (MIC) values are useful in guiding appropriate antimicrobial therapy however, routine provision and interpretation of MIC values to guide clinical decision-making is challenging.

**Objectives:**

This proof of concept study aims to demonstrate the clinical utility and application of Vitek^®^-derived MIC values through categorisation of clinical isolates as wild type.

**Method:**

A random selection of clinically relevant Gram negative isolates routinely tested on the Vitek^®^ instrument were included. The Vitek^®^ MIC values, for selected antimicrobials at the lowest calling range of that card, were compared to the broth microdilution reference method. The specified end-point was concordance between the two results if the reference MIC was less than or equal to the EUCAST-defined epidemiological cut-off value (ECOFF) for that drug-bug combination.

**Results:**

A total of 525 isolates were included (468 Enterobacterales and 57 *Pseudomonas aeruginosa*), with an overall concordance rate of 96.4% (508/525). Correct ECOFF categorisation by the Vitek^®^ was highest for ceftazidime and piperacillin (100%, *n* = 48 and *n* = 55, respectively) and lowest for cefepime (81.8%, *n* = 66).

**Conclusion:**

Vitek^®^-derived MIC values can be used to categorise organisms as wild-type if the MIC is reported at the card’s lowest calling range (≤) as there is high likelihood that the MIC is at or below the ECOFF. This has important implications for antimicrobial management, assisting in choice of agent and in improving probability of target attainment for desired pharmacodynamic targets which can translate into improved clinical outcomes.

**Contribution:**

Minimum inhibitory concentration data from an automated antimicrobial susceptibility testing instrument can be used to guide clinical decisions.

## Introduction

The importance of antimicrobial susceptibility testing (AST) in guiding appropriate antimicrobial therapy is highlighted by the need for dose optimisation to ensure that pharmacokinetic-pharmacodynamic (Pk-Pd) targets are achieved. The importance of the application of Pk-Pd targets in ensuring optimal use of antimicrobials is well-described;^1^ and in critically ill patients where dose optimisation is most critical, these targets are commonly not achieved.^2^ Furthermore, the argument that categorisation of an isolate as susceptible alone may not be sufficient to predict favourable clinical outcomes, and that more detailed susceptibility results are necessary is increasingly evident.^3,4^

A challenge arising from the need for tailored antimicrobial therapy, incorporating Pk-Pd data and minimum inhibitory concentration (MIC)-based susceptibility results, is the ability of routine diagnostics laboratories to provide sufficiently detailed AST results.^5^ Laboratories typically report categorical interpretations in the form of S (susceptible), I (intermediate [Clinical Laboratory Standards Institute {CLSI}] OR susceptible, increased exposure [European Committee on Antimicrobial Susceptibility Testing {EUCAST}]) or R (resistant). This limitation is often a consequence of methodology, where AST is largely performed using either the disk diffusion method or an automated system. The Vitek system (Biomerieux™, South Africa [SA]) is an automated system used by many diagnostic laboratories and provides MIC-based susceptibility results. There is, however, uncertainty amongst clinical microbiologists as to whether these MIC values should be reported and how to interpret them.

European Committee on Antimicrobial Susceptibility Testing (EUCAST) provides an epidemiological cut-off value (ECOFF) MIC which by definition distinguishes wild-type strains (devoid of any phenotypical acquired or mutational resistance mechanisms) from non-wild-type strains.^6^ Thus the ECOFF is an MIC value that sets a threshold for phenotypic assessment of the presence of resistance within an organism. It is distinct from clinical breakpoints which take into account pharmacological and clinical data, and is typically lower than clinical breakpoints for most ‘drug-bug’ combinations, hence a more sensitive marker of resistance. Given the inherent MIC reporting range limitations of automated systems, it may be more useful to evaluate the MIC values provided by these systems and compare them to the ECOFF value. From a clinical dose optimisation perspective, it has been suggested that the ECOFF could be used to guide antimicrobial dosing.^7^

The primary aim of this study is to provide proof of concept that Vitek^®^-derived MIC values can reliably be used as a correlate for an ECOFF, thereby distinguishing wild-type strains from non-wild-type strains. This information potentially provides clinically relevant guidance in terms of choice and dosing of antimicrobial therapy.

## Methods

A collection of Gram negative clinical isolates were utilised for this study. These non-duplicate clinical isolates were collected as part of various surveillance programmes spanning the years 2012–2019 and routinely tested on the Vitek^®^ 2 (Biomerieux™, SA) automated system. During this period, a variety of different Vitek cards were used, including Vitek cards N-255, N-256 and N-325. Isolate identification was confirmed on the Biomerieux™ MS MALDI-TOF and broth microdilution (BMD) as per the ISO standard^8^ was subsequently performed on all isolates.

The following antimicrobial agents, included in the BMD panel were used for comparative purposes on the basis of available ECOFFs (https://mic.eucast.org/search/): amikacin, ciprofloxacin, ceftazidime, cefepime, imipenem, meropenem, and piperacillin-tazobactam.

**FIGURE 1:**
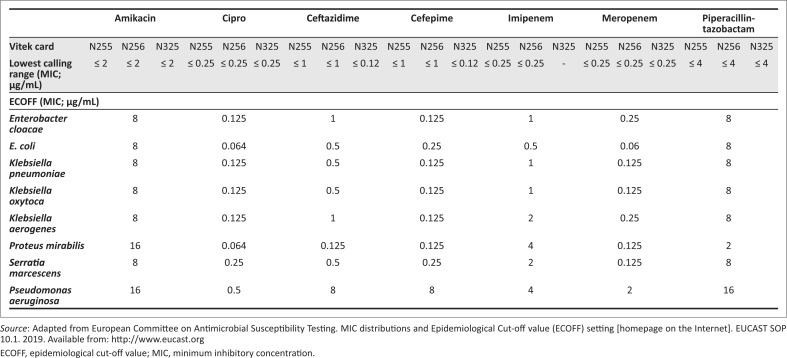
Vitek^®^ cards with lowest calling range and EUCAST-defined epidemiological cut-off values for study isolates.

The BMD MIC values were compared to those of the Vitek 2 AST results. For study, all isolates at the lowest calling range of that specific Vitek^®^ card, for each individual antimicrobial (i.e. those with MIC reported as ‘≤’) were deemed to have an MIC below the ECOFF. The BMD MIC values of these isolates were then compared to the respective EUCAST-derived ECOFF published for each specific ‘drug-bug’ combination (https://mic.eucast.org). Figure 1 highlights the respective calling range for each antimicrobial within the different Vitek^®^ cards, and the respective ECOFF for each organism. Concordance between the reported Vitek MIC and the ECOFF was then determined using the following assessment. If the BMD MIC value was less than or equal to the ECOFF value, it was deemed concordant and the Vitek card had correctly identified the isolate as a wild type for that specific antimicrobial.

## Results

A total of 525 isolates were included in the study consisting of 468 Enterobacterales isolates and 57 *Pseudomonas aeruginosa* isolates. The Enterobacterales isolates included: 76 *Enterobacter cloacae* complex species; 192 *E. coli*; 39 *Klebsiella aerogenes*; 39 *Klebsiella oxytoca*; 78 *Klebsiella pneumoniae*; 35 *Proteus mirabilis* and 9 *Serratia marcescens*.

The collective agreement between the BMD MIC value and Vitek^®^ MIC for correct ECOFF categorisation was 96.4% (508/525). Most antimicrobials demonstrated concordance of greater than 98.9% (349/353), with ceftazidime and piperacillin-tazobactam demonstrating the highest concordance (100% each), and cefepime the lowest concordance (81.8%).

Collectively, isolates with an MIC at the lowest calling range of the Vitek^®^ card for individual antimicrobials included: amikacin (*n* = 138); cefepime (*n* = 66); ceftazidime (*n* = 48); ciprofloxacin (*n* = 52); imipenem (*n* = 60); meropenem (*n* = 108) and piperacillin-tazobactam (*n* = 55). Stratification of organism per antimicrobial and the percentage agreement are reflected in Table 1.

**TABLE 1:** Overall concordance between Vitek^®^ lowest calling range result and epidemiological cut-off value with stratification of isolates tested per antimicrobial.

Antimicrobial	Isolates (*n*)			Isolates no. tested (% concordance)							
	Vitek MIC ≤	BMD MIC ≤ ECOFF	Overall concordance (%)	*E. coli*	*K. pneumoniae*	*K. oxytoca*	*K. aerogenes*	*P. mirabilis*	*E. cloacae*	*S. marcescens*	*P. aeruginosa*
Amikacin	138	136	98.6	45 (98)	31 (97)	8 (100)	8 (100)	8 (100)	16 (100)	2 (100)	18 (100)
Cefepime	66	54	81.8	24 (88)	9 (44)	2 (50)	6 (100)	7 (100)	11 (82)	3 (100)	4 (75)
Ceftazidime	48	48	100.0	21	3	2	6	2	10	1	3
Meropenem	108	104	96.3	34 (94)	20 (90)	7 (100)	9 (100)	9 (100)	16 (100)	2 (100)	11 (100)
Imipenem	60	59	98.3	31 (97)	10 (100)	4 (100)	2 (100)	1 (100)	11 (100)	0	1 (100)
Piperacillin-tazobactam	55	55	100.0	32	8	5	0	7	0	0	3
Ciprofloxacin	52	51	98.1	5 (100)	6 (100)	2 (100)	8 (100)	1 (100)	12 (92)	1 (100)	17 (100)

**Total**	**527**	**507**	**96.2**	**192**	**78**	**39**	**39**	**35**	**76**	**9**	**57**

BMD, broth microdilution; ECOFF, epidemiological cut-off value; MIC, minimum inhibitory concentration.

## Discussion

This study demonstrates a high concordance rate between the lowest calling range of commonly used antimicrobials in Vitek^®^ Gram negative cards and the associated ECOFF value for a variety of clinically relevant Gram negative pathogens. This is important in the context of improving antimicrobial management decisions where appropriate choice of agent based on probability of achieving the desired Pk-Pd target is influenced by the MIC of the pathogen. Given the biological variability intrinsic to AST methodology, an actual laboratory-derived MIC value is an approximation of the susceptibility of the isolate. It has been recommended that caution be exercised in using these values to guide targeted dose optimisation.^9^ However, achievement of Pk-Pd targets (C_max_:MIC; *f* T > MIC; AUC_24h_:MIC), which impact on clinical outcomes, is directly dependent on a pathogen’s MIC, with the likelihood thereof inversely proportional to the MIC.^2,10^ Thus, a reliable marker of a wild-type isolate with MICs within the ECOFF range is a clinically useful tool to guide both antimicrobial choice and dose.

Traditional reporting of susceptibility testing results according to categorisation (S/I/R) provides no indication as to the degree of susceptibility of the isolate. Clinical breakpoints are typically higher than the ECOFF and although breakpoints are linked to dosing recommendations which provide adequate drug exposure, in difficult-to-treat infections and critically ill patients, dose optimisation is challenging due to host-related factors. In these instances, the choice of an antimicrobial agent that is susceptible but has an MIC above the ECOFF increases the probability of microbiological and clinical failure by virtue of a lower probability of Pk-Pd target attainment. The post hoc analysis of microbial isolates from the MERINO trial illustrates this; notwithstanding the issues associated with piperacillin-tazobactam AST, the miscategorisation of resistant isolates as susceptible resulted in poorer outcomes, and a conclusion that required reassessment.^3^ This is in all likelihood a daily reality in clinical practise and also calls into question the results of clinical trial data for GNB infections.^11^ Routine susceptibility results that give guidance as to the susceptibility of an organism in relation to the ECOFF can potentially avert this situation and lead to improved antimicrobial use with resultant improved clinical outcomes. As the EUCAST steering committee expands its work and continues to provide ECOFF data on more organisms, the potential clinical utility becomes more of a reality. The clinical utility is largely unexplored and adds an additional dimension to the recently highlighted use of ECOFF.^12^ Epidemiological cut-off value zone sizes are provided by EUCAST and thus this could also be applied by laboratories that only use disk diffusion for AST, which is the recommended EUCAST method. This would require additional information to be provided on AST reports and concomitant education of clinicians as to the potential value thereof.

There are some notable limitations to this study. Firstly, only a limited number of Vitek^®^ cards and included antibiotics were tested; and thus, our results cannot be extrapolated to all currently available cards. Laboratories would be required to do their own validation based on the cards and antibiotics utilised. Secondly, although we tested a variety of common Gram negative clinical isolates, some were few in number and the correlation for some ‘drug-bug’ combinations would need to be investigated for a greater number of isolates. Additionally, further study on the wide variety of organisms not included in our study, particularly Gram positives, is warranted. We did not attempt to resolve discrepancies and investigate the possible reasons through repeat AST testing or molecular testing. This was on the basis of a proof of concept study using routine clinical isolates with reported susceptibility results, to assess the clinical utility of the Vitek^®^-derived MIC values irrespective of specifics of reported card limitations or ‘drug-bug’ combinations. Of interest for three meropenem discrepant results, two were OXA-48like positive isolates (MIC = 1 μg/mL by BMD) and one *E. coli* isolate had a meropenem MIC = 0.12 μg/mL as determined by BMD. The *E. coli* meropenem ECOFF was lowered in 2021 from 0.12 to 0.06 and considering the possibility of a two-fold dilution difference on either side of a measured MIC, it is plausible that the BMD result would be concordant on repeat testing. The OXA-48like phenotypes were detected based on reduced ertapenem susceptibility on the Vitek^®^, and with knowledge of the known genotype (isolates were genotypically confirmed), the meropenem MIC would be expected to fall outside of the ECOFF. Similarly, other phenotypic or genotypic characteristics of an isolate could be used to interrogate a Vitek^®^-derived MIC at the lower end of the calling range. This might be particularly important for cefepime, the antibiotic in our study demonstrating the lowest concordance rate (83.3%). However, again important to note is that five of the discrepant isolates had an MIC as determined by BMD, a single two-fold dilution above the ECOFF. Furthermore for two of the Vitek^®^ cards used in this study, the lowest calling range for cefepime is ≤ 1 μg/mL, a value well above the ECOFF for most of isolates tested in this study. This serves to highlight that manufacturers of automated susceptibility testing systems that provide MIC data need to consider inclusion of ECOFF values in the development process, and not only rely on inclusion of the susceptible clinical breakpoint.

## Conclusion

In summary, we provide proof of concept that Vitek^®^-derived MIC values can be used to guide clinically appropriate antimicrobial management decisions. Further evaluation of more ‘drug-bug’ combinations and different Vitek^®^ cards is warranted to determine the generalisability and broader applicability of these results.
